# The mTOR/PGC-1α/SIRT3 Pathway Drives Reductive Glutamine Metabolism to Reduce Oxidative Stress Caused by ISKNV in CPB Cells

**DOI:** 10.1128/spectrum.02310-21

**Published:** 2022-01-12

**Authors:** Xiaozhe Fu, Kejin Li, Yinjie Niu, Qiang Lin, Hongru Liang, Xia Luo, Lihui Liu, Ningqiu Li

**Affiliations:** a Pearl River Fisheries Research Institute, Chinese Academy of Fishery Sciences, Key Laboratory of Fishery Drug Development, Ministry of Agriculture, Guangdong Province Key Laboratory of Aquatic Animal Immune Technology, Guangzhou, China; Oklahoma State University, College of Veterinary Medicine

**Keywords:** ISKNV, reductive glutamine metabolism, mTOR, SIRT3, PGC-1α

## Abstract

Under oxidative stress, viruses prefer glycolysis as an ATP source, and glutamine is required as an anaplerotic substrate to replenish the TCA cycle. Infectious spleen and kidney necrosis virus (ISKNV) induces reductive glutamine metabolism in the host cells. Here we report that ISKNV infection the increased NAD+/NADH ratio and the gene expression of glutaminase 1 (GLS1), glutamate dehydrogenase (GDH), and isocitrate dehydrogenase (IDH2) resulted in the phosphorylation and activation of mammalian target of rapamycin (mTOR) in CPB cells. Inhibition of mTOR signaling attenuates ISKNV-induced the upregulation of GLS1, GDH, and IDH2 genes expression, and exhibits significant antiviral activity. Moreover, the expression of silent information regulation 2 homolog 3 (SIRT3) in mRNA level is increased to enhance the reductive glutamine metabolism in ISKNV-infected cells. And those were verified by the expression levels of metabolic genes and the activities of metabolic enzymes in SIRT3-overexpressed or SIRT3-knocked down cells. Remarkably, activation of mTOR signaling upregulates the expression of the peroxisome proliferator-activated receptor γ coactivator-1α (PGC-1α) gene, leading to increased expression of SIRT3 and metabolic genes. These results indicate that mTOR signaling manipulates reductive glutamine metabolism in ISKNV-infected cells through PGC-1α-dependent regulation of SIRT3. Our findings reveal new insights on ISKNV–host interactions and will contribute new cellular targets to antiviral therapy.

**IMPORTANCE** Infectious spleen and kidney necrosis virus (ISKNV) is the causative agent of farmed fish disease that has caused huge economic losses in fresh and marine fish aquaculture. The redox state of cells is shaped by virus into a favorable microenvironment for virus replication and proliferation. Our previous study demonstrated that ISKNV replication induced glutamine metabolism reprogramming, and it is necessary for the ISKNV multiplication. In this study, the mechanistic link between the mTOR/PGC-1α/SIRT3 pathway and reductive glutamine metabolism in the ISKNV-infected cells was provided, which will contribute new insights into the pathogenesis of ISKNV and antiviral treatment strategies.

## INTRODUCTION

Many studies have shown that different viruses can induce oxidative stress (OS) via interfering with the reduction/oxidation (redox) homeostasis of the host cells (1). The redox state of cells is shaped by virus into a favorable microenvironment for virus replication and proliferation (2). Viruses alter mitochondrion metabolism to compensate for the energy demands for their own replication and proliferation in cells (3). Similar to tumor cells, viruses prefer glycolysis as an ATP source under oxidative stress although less energy-efficient than oxidative phosphorylation, while glutamine is required as an anaplerotic substrate to replenish the tricarboxylic acid (TCA) cycle to synthesize fatty acid for virus amplification (4). Glutamine is one of the most abundant cellular amino acids and it is necessary for the generation of energy, macromolecules, and second messengers in cells (5). Glutamine is converted to glutamate by glutaminase 1 (GLS1), and then glutamate dehydrogenase (GDH) catalyzes the conversion of glutamate to α-ketoglutarate (α-KG), which enters the TCA cycle through an oxidative pathway mediated by α-ketoglutarate dehydrogenase (α-KGDH). Alternatively, α-ketoglutarate can be reductively carboxylated to citrate for lipogenesis through the reductive carboxylation pathway (reductive glutamine metabolism) mediated by isocitrate dehydrogenase 1 and 2 (IDH1 and IDH2) (6–9). It has been proved that reductive glutamine metabolism occurs in a small cohort of cells to support redox homeostasis and synthesis of lipids, nucleotides, and urea (10).

Infectious spleen and kidney necrosis virus (ISKNV) is the causative agent of farmed fish disease that has caused huge economic losses in fresh and marine fish aquaculture (11, 12). It was reported that ISKNV ORF005 located in the inner mitochondrial membrane induced the decrease of mitochondrial membrane potential, resulting in oxidative stress through mitochondrial respiration (13). He et al. reported that ISKNV infection induced high reduced/oxidized glutathione ratio leading to changes of redox state (14). Previously, we demonstrated that ISKNV replication induced aerobic glycolysis (15), and glutamine metabolism is necessary for the ISKNV multiplication (16). Metabolomics analysis revealed that ISKNV-infected cells preferred to utilize glutamine to synthetize lipid for ISKNV maturation at the late stage of infection (17). Further study in our lab found that ISKNV infection improved the carbon flux of the reductive pathway. through analysis of transcriptome, proteome, metabolomics, and furthermore IDH2-mediated reductive glutamine metabolism played an important role in the proliferation of ISKNV (data not shown). However, the mechanism of ISKNV-induced reductive glutamine metabolism remains to be clarified.

Mammalian target of rapamycin (mTOR) regulates protein synthesis, glucose metabolism, lipid metabolism, and other life processes by phosphorylating downstream effectors (18, 19). It has also been determined that the activation of the mTOR pathway promotes glutamine metabolism in cancer cells (20, 21). Some studies have found that viral infection can activate the mTOR pathway and manipulate glycolysis or lipid metabolism in the host cells, such as hepatitis B virus (HBV) (22), white spot syndrome virus (WSSV) (23), and human herpesvirus 6 (HHV-6) (24). Until now, only avian reovirus (ARV) has been proved to induce glutamine metabolism by activating the mTOR pathway (25).

Sirtuins (SIRTs) are NAD^+^-dependent deacetylases that participate in cell signaling transduction involved in the cell cycle, mitochondrial aging, energy production, and cellular metabolism (26, 27). SIRT3, one kind of SIRT, is a key regulator of cellular metabolic status (28, 29). Previous studies confirmed that SIRT3 regulates a wide range of metabolic pathways involved in the cancer progression, and responds to nutrient deficiency (30–33). It has been reported that GDH and IDH2 can be activated as the downstream target metabolic enzyme of SIRT3 (34). Peroxisome proliferator-activated receptor γ coactivator-1α (PGC-1α) is a transcriptional coactivator that coordinates the expression of energy metabolism and mitochondrial homeostasis related genes, including SIRT3 (35) and IDH2 (36). Recently, a study reported that the inhibition of the mTOR pathway strikingly decreased the expression of the PGC-1α gene in alveolar epithelial cells, indicating that PGC-1α is a downstream effector of mTOR (37). To illustrate the mechanism of ISKNV-induced reductive glutamine metabolism, we first investigated relationships between the activation of mTOR and enhanced reductive glutamine metabolism induced by ISKNV. Then, functions of PGC-1α and SIRT3 were determined during this process. The data presented here provide a mechanistic link between the mTOR/PGC-1α/SIRT3 pathway and reductive glutamine metabolism in the ISKNV-infected cells, and will contribute new insights to the pathogenesis of ISKNV and antiviral treatment strategies.

## RESULTS

### ISKNV increased NAD+/NADH ratio and regulated GLS1, GDH, and IDH2 via mTOR.

**FIG 1:**
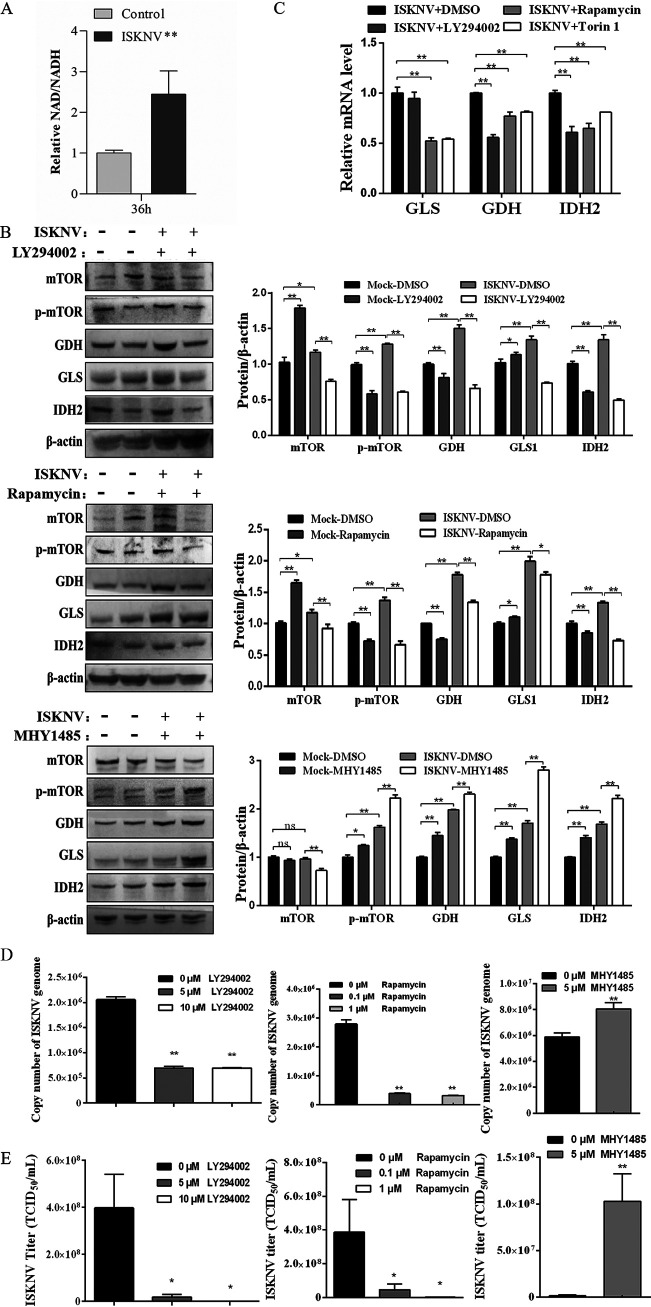
ISKNV infection regulated expression of GLS1, GDH, and IDH2 via mTOR. (A) NAD+/NADH ratio in ISKNV-infected and non-infected cells. (B) mRNA expression of GLS1, GDH, and IDH2 in ISKNV-infected and non-infected cells treated with 10 μM LY294002, 1 μM rapamycin, or 5 μM MHY1485 for 72 h detected by real-time PCR. (C) Protein expression of mTOR, p-mTOR, GLS1, GDH, and IDH2 in ISKNV-infected and non-infected cells treated with 10 μM LY294002, 1 μM rapamycin, or 5 μM MHY1485 for 72 h determined by Western blotting. (D) The copy number of ISKNV genome in infected cells treated with LY294002, rapamycin, or MHY1485 for 36 h by determination of real-time PCR. (E) ISKNV titers in supernatant of infected cells treated with LY294002, rapamycin, or MHY1485 for 72 h by TCID_50_ analysis.

It has been reported that NAD+/NADH plays an important role in reductive glutamine metabolism to protect cells from oxidative stress and support redox homeostasis (10). Thus, we first detected the NAD+/NADH ratio in ISKNV-infected cells. The result showed that ISKNV infection increased [NAD+] and decreased [NADH] compared to the control group (Fig. 1A).

Then we determined the expression change of major enzymes in the reductive glutamine metabolism pathway. Our previous study determined the working concentration and inhibitory efficacy of LY294002 (an inhibitor of PI3K) and rapamycin (an inhibitor of mTOR) (38). The activatory efficacy of MHY1485 (a specific activator of mTOR) was also proved in this study, and the working concentration was 0.05 μM (data not shown). As shown in Fig. 1B, p-mTOR protein expression remarkably decreased in CPB cells treated with LY294002 or rapamycin and increased in CPB cells treated with MHY1485, suggesting that LY294002 and rapamycin blocked the activation of mTOR signaling and MHY1485 activated the mTOR signaling. It was also found that ISKNV infection significantly promoted expression of GLS1, GDH, and IDH2 in protein level compared to those of the control group. But expression of GLS1, GDH, and IDH2 in protein level significantly decreased by inhibiting mTOR signaling with LY294002 and rapamycin or increased by activating mTOR signaling with MHY1485 in the ISKNV-infected cells compared to the control group. Similar change about expression of GLS1, GDH, and IDH2 genes was observed in the ISKNV-infected cells with the same treatment (Fig. 1C).

We further assessed the effects of mTOR on replication and proliferation of ISKNV. It was found that that copy number of the ISKNV genome was significantly decreased when inhibiting mTOR with LY294002 or rapamycin, but significantly increased when activating mTOR with MHY1485 in a concentration-dependent manner (Fig. 1D). The results of ISKNV progeny production also showed that titers of ISKNV were decreased by treatment with LY294002 or rapamycin, and significantly increased by treatment with MHY1485 in a concentration-depended manner (Fig. 1E). All of the above results indicated that mTOR played a positive regulatory role in the reductive glutamine metabolism and ISKNV proliferation.

### ISKNV upregulated expression of SIRT3 to induce reductive glutamine metabolism via mTOR.

**FIG 2:**
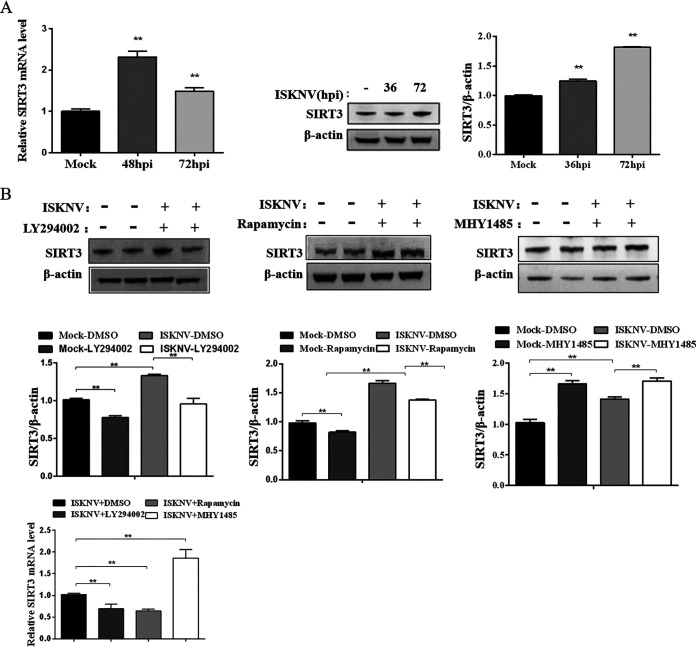
ISKNV infection upregulated expression of SIRT3 via mTOR. (A) mRNA and protein expression of SIRT3 in ISKNV-infected or noninfected cells at 36 hpi and 72 hpi detected by real-time PCR and Western blotting. (B) Protein and mRNA expression of SIRT3 in ISKNV-infected and non-infected cells treated with 10 μM LY294002, 1 μM rapamycin, or 5 μM MHY1485 at 72 hpi determined by Western blotting and real-time PCR.

To investigate the role of SIRT3 in the regulation of metabolism, we first examined the expression of SIRT3 in the ISKNV-infected cells. Results showed that expression of SIRT3 in mRNA and protein levels significantly increased at 36 hours postinfection (hpi) and 72 hpi, respectively (Fig. 2A). Next, inhibiting mTOR signaling with LY294002 or rapamycin significantly decreased expression of SIRT3 in mRNA and protein levels, but activating mTOR signaling with MHY1485 increased expression of SIRT3 in mRNA and protein levels in the ISKNV-infected cells compared to the control group (Fig. 2B).

**FIG 3:**
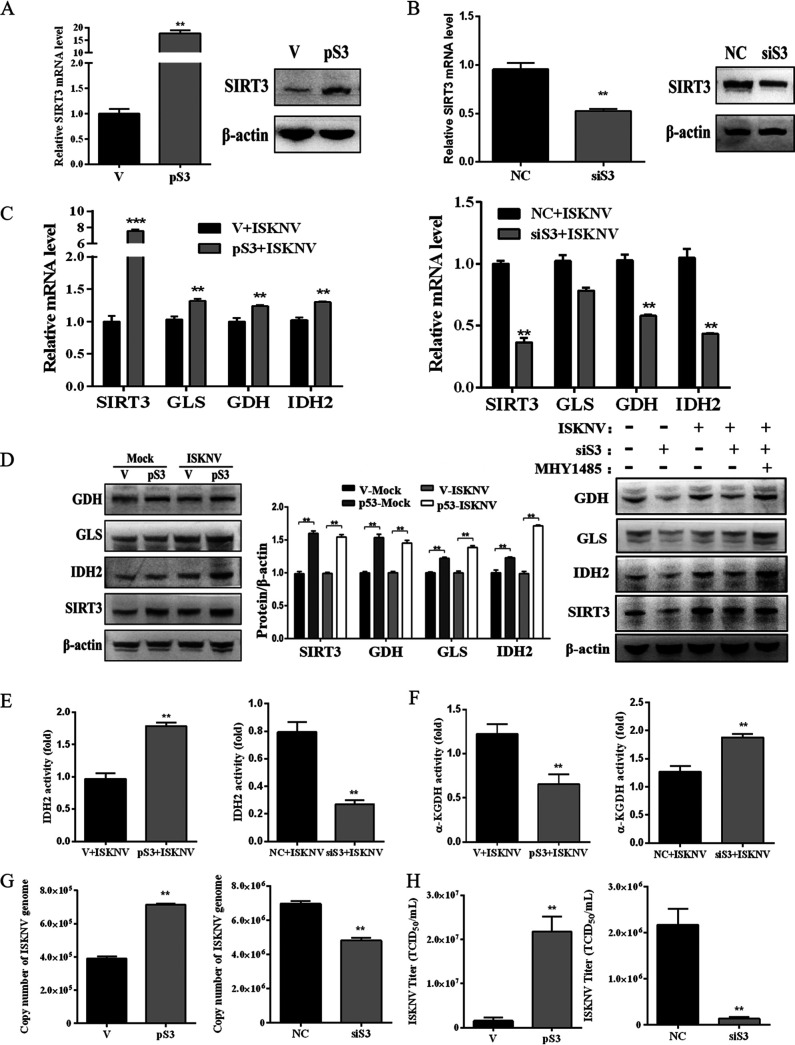
ISKNV infection enhanced reductive glutamine metabolism via the mTOR/SIRT3 pathway. (A–B) CPB cells were transfected with empty vector (V) or plasmid pCMV-SIRT3 (pS3) to construct a stable SIRT3-overexpressed cell line. CPB cells were transfected with nontargeting siRNAs (NC) or siRNAs target SIRT3 gene (siS3) to knock down the expression of SIRT3. mRNA and protein expression of SIRT3 in V cells, pS3 cells, NC cells, or siS3 cells determined by real-time PCR and Western blotting. (C) mRNA expression of SIRT3, GLS1, GDH, and IDH2 in transfected cells infected or noninfected with ISKNV. (D) Protein expression of SIRT3, GLS1, GDH, and IDH2 in pS3 or siS3 cells infected or noninfected with ISKNV detected by Western blotting. (E) IDH2 activity at 72 hpi in pS3 or siS3 cells infected with ISKNV. (F) α-KGDH at 72 hpi in pS3 or siS3 cells infected with ISKNV. (G) The copy number of ISKNV genome in pS3 or siS3 cells at 36 hpi by determination of real-time PCR. (H) ISKNV titers in supernatant of pS3 or siS3 cells at 72 hpi by TCID_50_ analysis.

Subsequently, we established a stable overexpressed SIRT3 cell line by transfection of pCMV-SIRT3 plasmid, designated as pS3 cells. And a SIRT3-knocked-down cell line was also constructed by transfection of specific siRNAs, designated as siS3 cells. Efficacy of overexpression and knock-down of SIRT3 was identified by qRT-PCR and Western blotting. As shown in Fig. 3A and B, expression of SIRT3 in mRNA and protein levels increased significantly in pS3 cells and decreased significantly in siS3 cells. Then, we investigated glutamine reductive metabolism regulation by SIRT3. Results showed that expression of GLS1, GDH, and IDH2 in mRNA levels significantly increased in pS3 cells or decreased in siS3 cells infected with ISKNV compared to the mock group (Fig. 3C). The similar changes of expression were also observed in protein level (Fig. 3D). Furthermore, activation of mTOR with MHY1485 increased the expression of SIRT3, GLS1, GDH, and IDH2 in protein level in siS3cells infected with ISKNV (Fig. 3D).

SIRT3 can regulate enzyme activity through deacetylation. Therefore, we measured the effect of SIRT3 on IDH2 activity, a key metabolic enzyme in the reductive pathway of glutamine, and α-KGDH activity, a key metabolic enzyme in the oxidative pathway of glutamine. Results showed that IDH2 activity significantly increased in pS3 cells infected with ISKNV, and significantly decreased in siS3 cells infected with ISKNV (Fig. 3E). However, α-KGDH activity significantly decreased in pS3 cells infected with ISKNV, and significantly increased in siS3 cells infected with ISKNV (Fig. 3F).

Meanwhile, we assessed the effect of SIRT3 on the replication and proliferation of ISKNV. It was found that copy numbers of ISKNV genome significantly increased in SIRT3-overexpressed cells infected with ISKNV, but remarkably decreased in SIRT3-knockdown cells infected with ISKNV (Fig. 3G). The results of ISKNV progeny production also showed that titers of ISKNV significantly increased in SIRT3-overexpressed cells and decreased in SIRT3-knockdown cells (Fig. 3H). All of the above results indicated that ISKNV infection promoted reductive glutamine metabolism by activating an mTOR/SIRT3 pathway.

### PGC-1α regulated expression of SIRT3 and metabolic-related genes and benefited the production of ISKNV.

**FIG 4:**
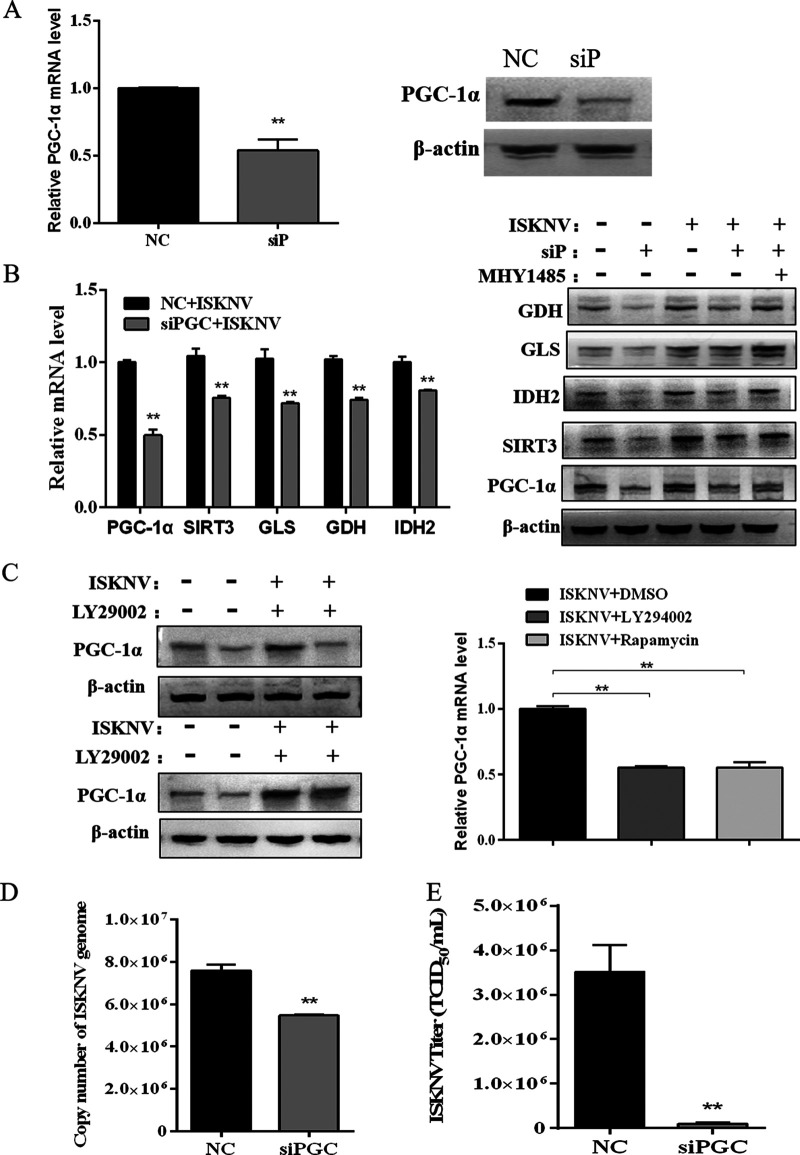
PGC-1α regulated expression of SIRT3 and metabolic genes, and benefited the production of ISKNV. (A) CPB cells were transfected with non-targeting siRNAs (NC) or siRNAs target PGC-1α gene (siP) to knock down the expression of PGC-1α. mRNA and protein expressions of PGC-1α in NC and siP cells were detected by real-time PCR and Western blotting. (B) mRNA and protein expression of PGC-1α, SIRT3, GLS1, GDH, and IDH2 in NC and siP cells infected or noninfected with ISKNV, determined by real-time PCR and Western blotting. (C) mRNA and protein expression of PGC-1α in ISKNV-infected or noninfected CPB cells treated with LY294002, rapamycin, or MHY1485 for 72 h detected by real-time PCR and Western blotting. (D) The copy number of the ISKNV genome in NC or siP cells at 36 hpi by determination of real-time PCR. (E) ISKNV titers in supernatant of NC or siP cells at 72 hpi by TCID_50_ analysis.

**FIG 5:**
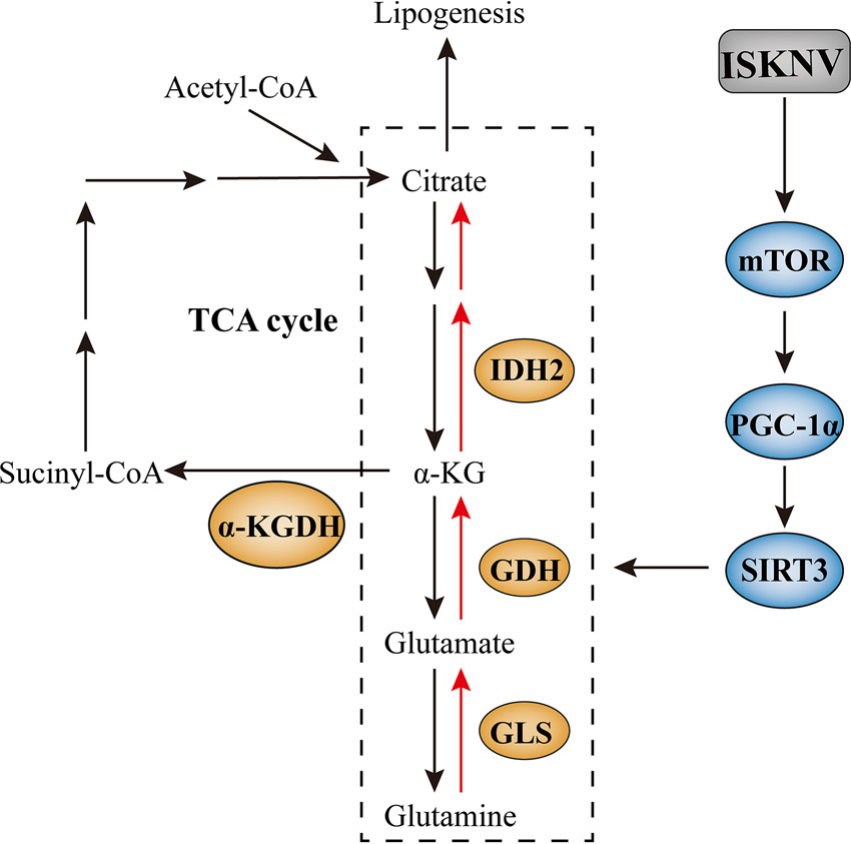
Proposed model of how ISKNV-induced reductive glutamine metabolism was regulated by the mTOR/PGC-1α/SIRT3 pathway. The reductive glutamine metabolism pathway (red arrows) enhanced at 72 hpi. ISKNV infection activated the mTOR signaling and upregulated the expression of PGC-1α and SIRT3 genes, leading to the reductive glutamine metabolism enhanced (blue), as evidenced by the gene expression of enzymes and the activities of enzymes (orange). Metabolic genes expression data are compiled from the present study and our previous study.

PGC-1α is a transcription coactivator and downstream effector of mTOR. To investigate whether PGC-1α is involved in the regulation of metabolic-related gene expression, we knocked down the PGC-1α gene in CPB cells using specific siRNAs, designated as siP cells (Fig. 4A). As shown in Fig. 4B, expression of SIRT3, GLS1, GDH, and IDH2 in mRNA and protein levels decreased in the siP cells infected with ISKNV compared to the control group. But activation of mTOR signaling with MHY1485 increased the expression of PGC-1α, SIRT3, GLS1, GDH, and IDH2 in protein levels in siP cells infected with ISKNV (Fig. 4B). Results also showed that expression of PGC-1α in mRNA and protein levels decreased by inhibiting mTOR signaling with LY294002 or rapamycin in the ISKNV-infected or uninfected cells (Fig. 4C). Moreover, the copy number of ISKNV genome and titers of ISKNV was reduced in PGC-1α-knockdown cells, suggesting that PGC-1α benefited ISKNV replication and proliferation (Fig. 4D and E). All of the above results indicated that ISKNV infection induced reductive glutamine metabolism via the mTOR/PGC-1α/SIRT3 pathway (Fig. 5).

## DISCUSSION

Viruses have evolved to manipulate host cellular metabolism to support their energetic and biosynthetic requirements for successful replication by different pathways (4, 39), such as inducing OS in infected cells, providing a favorable microenvironment for virus replication at the early stage of the ISKNV infection cycle (2). Reductive glutamine metabolism produces citrate that is converted to fatty acid and supplements lipid pools via the lipid biosynthesis pathway (9, 40). Lipids are the major components of biological membranes and necessary for the viral maturation. Thus, enhanced reductive glutamine metabolism provided the raw material for the viral maturation at the late stage of the ISKNV infection cycle.

As the main mitochondrial deacetylase, Sirt3 plays a key role in energy metabolism and oxidative stress (41, 42). Our results showed that SIRT3 positively regulated IDH2 activity and negatively regulated α-KGDH activity in ISKNV-infected cells, which indicated SIRT3 could regulate reductive glutamine metabolism. It was reported that SIRT3 blocked HBV-induced oxidative stress and cell damage (43), and SIRT3 increased the level of NADPH and the ratio of oxidized glutathione that protect cells from oxidative damage (33). It is well known that NADPH is one of the products of reductive glutamine metabolism. It was also reported that SIRT3 promoted reductive glutamine metabolism in cancer cells through deacetylation and activation of IDH2 (34). Furthermore, the IDH2-mediated reductive pathway and the α-KGDH-mediated oxidative pathway are antagonistic and regulated by SIRT3-mediated deacetylation (44). Therefore, we presume that SIRT3-mediated reductive glutamine metabolism may play a role in antioxidant stress and contribute to infected-cells survival, which benefits the production of high-titer stocks of ISKNV.

The role of mTOR is crucial in regulating mitochondrial functions and promoting metabolism (45). The mTOR signaling pathway is regulated by its upstream factor PI3K/Akt. Thus, in this study the activator of PI3K was used for activating mTOR. Our results showed that mTOR played a positive regulatory role in the reductive glutamine metabolism in ISKNV infected cells through regulating expression of GLS1, GDH, and IDH2. Previous studies also showed that mTORC1 promoted glutamine anaplerosis by activating glutamate dehydrogenase (GDH) and positively regulates GLS (20, 21). PGC-1s play a central role in regulating mitochondrial functions and metabolism (46). Our results showed PGC-1α positively regulated the reductive glutamine metabolism in ISKNV infected cells. In glutamine metabolism, PGC-1α increases the expression of glutamine metabolism genes, and elevates glutamine-mediated lipogenesis in hypoxia (46). It has been reported that mTOR activated many downstream factors through phosphorylation, such as CREB (47). Furthermore, the CREB/PGC-1α pathway contributed to a protective effect of fucoxanthin on oxidative damage (48). Besides, a previous study revealed that PGC-1α/ERRα signaling could activate the SIRT3 promoter (49). Thus, we presumed ISKNV induced the reductive glutamine metabolism via activating mTOR/PGC-1α/SIRT3 signaling. Also, our results also illustrated that PGC-1α was positively regulated by mTOR and PGC-1α positively regulated expression of SIRT3 and metabolic-related genes in ISKNV-infected cells.

In conclusion, mTOR the signaling pathway manipulated reductive glutamine metabolism to reduce oxidative stress in ISKNV-infected cells through PGC-1α-dependent regulation of SIRT3. Our findings revealed new insights into ISKNV-host interactions and provided novel potential cellular targets for antiviral therapy.

## MATERIALS AND METHODS

### Cell culture, virus infection, and treatment of biochemical drugs.

The Chinese perch brain (CPB) cells were maintained at 28°C in Leibovitz's L-15 medium (Gibco, USA) containing 10% fetal bovine serum (FBS) (Gibco, USA) (50). ISKNV was isolated previously and propagated in CPB cells at 28°C (50). LY294002, rapamycin, and MHY1485 (Sigma-Aldrich, USA) were solubilized in dimethyl sulfoxide (DMSO) to the different stock solutions. Then these solutions were diluted with Leibovitz's L-15 medium containing 2% FBS and added to the CPB cells at the working concentration of LY294002 (10 μM), rapamycin (1 μM), or MHY1485 (0.05 μM).

### Sampling.

CPB cells were incubated with ISKNV (MOI = 1) for 2 h, then the supernatants were removed. They were fed medium containing 5% FBS and the working concentration of LY294002, rapamycin, or MHY1485. Cells or supernatants were harvested at 36 h and 72 h postinfection (hpi) and used for qPCR, Western blotting, and viral titration detection. Titers of ISKNV were determined by TCID_50_ assay using the Karber method (51). Uninfected cells maintained with medium containing DMSO were used as control.

### Quantitative PCR assay.

Total RNAs were extracted from ISKNV-infected or un-infected cells with TRIzol reagent (Invitrogen, USA) and synthesized into cDNA using 5 × All-In-One RT MasterMix Kit (Abm, Guangzhou, China) as described previously (15). The qPCR was performed in an ABI 7500 real-time Detection System (Applied Biosystems, USA) using SYBR Premix Ex Taq kit (TaKaRa, Japan) as described previously (52). All reactions were repeated three times, and the dissociation curve was analyzed after each assay to determine target specificity. The relative expression ratio of gene mRNA was determined by the formula 2^-ΔΔCT^ with 18S rRNA as the internal control.

The quantification of ISKNV copies was determined as described previously (53). Briefly, the supernatants of ISKNV-infected CPB cells were treated with a final concentration of 160 μg/mL Proteinase K (Promega, USA) for 2 h at 56°C, then inactivated at 95°C for 10 min and centrifuged at 10,000 rpm for 10 min. The centrifuged supernatants were used for q-PCR determination by using Premix Ex TaqTM assay (TaKaRa, China) carried out in an ABI 7500 real-time Detection System (Applied Biosystems, USA). All specific primers and probes are listed in Table 1.

**TABLE 1:** Primers used in this study

Name	Sequence (5′–3′)	Application
ORF007-F	CGAGGCCACATCCAACATC	quantify ISKNV
ORF007-R	CGCCTTTAACGTGGGATATATTG	
ORF007-Probe	FAM-CACCAAACTGACCGCGGACTCGT	
18-F	CATTCGTATTGTGCCGCTAGA	internal control
18S-R	CAAATGCTTTCGCTTTGGTC	
q-GLS1-F	TCCTGCGGCATGTACGACTTCT	gene expression
q-GLS1-R	CCAGCTTGTCCAGTGGAGGTGA	
q-GDH-F	AGGTCCGTCACTATGCCGATGC	
q-GDH-R	AGATCCTCCACCAGCTTGTCCTC	
q-IDH2-F	GTCATCAGTGTGGTCACGGTACG	
q-IDH2-R	TGGAGATGGACGGAGACGAGATG	
q-α-KGDH-F	TCCAGCCAGGCGTAGTACATCTC	
q-α-KGDH-R	CCAGCAGCGGCCAATCACAG	
q-SIRT3-F	AGCCACCGACCCAACTACATAC	
q-SIRT3-R	GTGTAGCACAGGTGACAGGAAGC	
q-PGC-1α-F	TTGCCACTTCCTTTGACCC	
q-PGC-1α-R	CTGGGGTTCCAGCAATCTC	
SIRT3-F	ATGAACAGGTCCAGGTCCAGCTGGG	cloning SIRT3
SIRT3-R	TCAGTTGCTCAGGCTGGATGATGCAG	

### Western blotting.

The total proteins were extracted from cell samples using RIPA lysate buffer containing PMSF and phosphatase inhibitor. The Pierce BCA protein assay kit (Thermo Fisher Scientific, Germany) was used to quantify proteins. Equal amounts of proteins were separated by SDS-PAGE, using 7% polyacrylamide gels and then transferred onto PVDF membranes (Millipore, USA). The membranes were blocked with 1 × TBS buffer containing 5% BSA and 0.05% Tween 20 for 3 h, incubated with primary antibodies at appropriate dilutions for 2 h and subsequently incubated with peroxidase-conjugated goat-anti-rabbit IgG (1:5000 dilutions) for 1 h. Immunoreactive proteins were visualized by using DAB staining (Beyotime, China). Primary antibodies used in this study included anti-mTOR (7C10), anti-p-mTOR (ser2448) (Cell Signaling Technology, Danvers, MA), anti-SIRT3, anti-GLS1, anti-GDH, anti-IDH2, anti-PGC-1α, anti-α-KGDH (Proteintech, USA), and anti-β-actin (Abcam, USA).

### Establishment of SIRT3 overexpression stable cell lines and siRNA transfection.

Mandarin fish (*Siniperca chuatsi*) SIRT3 gene was cloned into eukaryotic expression vector pCMV-SV40 designated as pCMV-SIRT3 (Kan R). Primers used to clone SIRT3 gene are listed in Table 1. CPB cells were transfected with pCMV-SIRT3 plasmid using FuGENE6 transfection reagent (Promega, USA), and then were selected by G418 to obtain a stable SIRT3-overexpressed cell line. SIRT3 siRNA, PGC-1α siRNA and scrambled siRNA were synthesized by Wuhan GeneCreate Biological Engineering Co. Ltd. The siRNA sequences are listed in Table 2. CPB cells were transfected with 50 nM siRNA using transfection reagent EL (TransGen, China). Then, the supernatant was removed and replaced by fresh medium at 4 h posttransfection. The expression of the target gene was detected by qPCR and Western blotting at 72 h posttransfection.

**TABLE 2:** siRNA sequences used in this study

Name	Sequence (5′–3′)	Application
siRNA-SIRT3-S	CCCAAAGCAGACCUGCUAAdTdT	
siRNA-SIRT3-AS	UUAGCAGGUCUGCUUUGGGdTdT	
siRNA-PGC-1α-S	CGGUGUGGAGAGAAUUAGAdTdT	gene silencing
siRNA-PGC-1α-AS	UCUAAUUCUCUCCACACCGdTdT	
scrambled siRNA-S	UUCUCCGAACGUGUCACGUdTdT	
scrambled siRNA-AS	ACGUGACACGUUCGGAGAAdTdT	gnegative control

### Enzyme activity assay.

The Mitochondria Isolation Kit for Cultured Cells (Thermo Fisher Scientific, Germany) was used to extract mitochondria of ISKNV-infected or uninfected cells. An Isocitrate Dehydrogenase Activity Assay Kit (Sigma-Aldrich, Germany) and an α-Ketoglutarate Dehydrogenase Activity Colorimetric Assay Kit (Sigma-Aldrich, Germany) were used to detect the enzyme activity in cells. Briefly, mitochondria from 5 × 10^6^ cells were homogenized in 300 μL of assay buffer to prepare sample solution. Twenty μL of sample solution was pipetted into the wells of a 96-well plate. Then, a reaction mixture (8 μL developer, and 2 μL α-KGDH substrate or 2 μL IDH substrate) was added to each well. For IDH2 activity analysis, 2 μL of NADP+ was added additionally to the reaction mixture. After 3 min, the absorbance at 450 nm was measured every minute in a microplate reader (Infinite M200 Pro, Tecan, Switzerland) until the value of the most active sample is greater than the value of the highest standard. IDH2 activity was defined as the production of 1.0 μM NADPH per minute. α-KGDH activity was defined as the production of 1.0 μM NADH per minute. Three replicates of each sample were performed. For samples exhibiting significant background, we included a sample blank by omitting the substrate.

### NAD+/NADH ratio measurement.

NAD+/NADH ratio was measured in cell homogenates using the NAD/NADH Quantitation Kit (Sigma, USA) according to the manufacturer's instructions, respectively. The NAD+/NADH ratio was normalized to the protein concentration of the homogenate determined using the Bradford method. CPB cells infected with ISKNV at 36 hpi were sampled for measure.

### Statistical analysis.

Data were originated from three repeated experiments and presented as Mean ± SD. Student's *t* test or one-way analysis of variance (ANOVA) was performed with SPSS software (version 21.0) and used to compare the data differences between groups. * denotes *P* < 0.05, ** denotes *P* < 0.01, ns denotes nonsignificant in Fig. 1–4.
